# BARD1 serum autoantibodies for the detection of lung cancer

**DOI:** 10.1371/journal.pone.0182356

**Published:** 2017-08-07

**Authors:** Maxim Pilyugin, Pascaline Descloux, Pierre-Alain André, Viktoria Laszlo, Balazs Dome, Balazs Hegedus, Sylvain Sardy, Samuel Janes, Andrea Bianco, Geoffrey J. Laurent, Irmgard Irminger-Finger

**Affiliations:** 1 Molecular Gynecology and Obstetrics Laboratory, Department of Gynecology and Obstetrics, Medical Genetics and Laboratories, Geneva University Hospitals, Geneva, Switzerland; 2 Departement of Mathematics, University of Geneva, Geneva, Switzerland; 3 Division of Thoracic Surgery, Department of Surgery, Comprehensive Cancer Center Vienna, Medical University of Vienna, Vienna, Austria; 4 National Koranyi Institute of Pulmonology, Budapest, Hungary; 5 Department of Thoracic Surgery, National Institute of Oncology-Semmelweis University, Budapest, Hungary; 6 Molecular Oncology Research Group, Hungarian Academy of Sciences-Semmelweis University, Budapest, Hunagary; 7 Lungs for Living Research Centre, UCL Respiratory, Rayne Institute, University College London, London, United Kingdom; 8 Dipartimento di Medicina e Scienze della Salute “V. Tiberio”, Università del Molise, Campobasso, Italy; 9 Institute for Respiratory Health, University of Western Australia and Harry Perkins Institute of Medical Research, Perth, Australia; University of Nebraska Medical Center, UNITED STATES

## Abstract

**Purpose:**

Currently the screening for lung cancer for risk groups is based on Computed Tomography (CT) or low dose CT (LDCT); however, the lung cancer death rate has not decreased significantly with people undergoing LDCT. We aimed to develop a simple reliable blood test for early detection of all types of lung cancer based on the immunogenicity of aberrant forms of BARD1 that are specifically upregulated in lung cancer.

**Methods:**

ELISA assays were performed with a panel of BARD1 epitopes to detect serum levels of antibodies against BARD1 epitopes. We tested 194 blood samples from healthy donors and lung cancer patients with a panel of 40 BARD1 antigens. Using fitted Lasso logistic regression we determined the optimal combination of BARD1 antigens to be used in ELISA for discriminating lung cancer from healthy controls. Random selection of samples for training sets or validations sets was applied to validate the accuracy of our test.

**Results:**

Fitted Lasso logistic regression models predict high accuracy of the BARD1 autoimmune antibody test with an AUC = 0.96. Validation in independent samples provided and AUC = 0.86 and identical AUCs were obtained for combined stages 1–3 and late stage 4 lung cancers. The BARD1 antibody test is highly specific for lung cancer and not breast or ovarian cancer.

**Conclusion:**

The BARD1 lung cancer test shows higher sensitivity and specificity than previously published blood tests for lung cancer detection and/or diagnosis or CT scans, and it could detect all types and all stages of lung cancer. This BARD1 lung cancer test could therefore be further developed as i) screening test for early detection of lung cancers in high-risk groups, and ii) diagnostic aid in complementing CT scan.

## Introduction

Lung cancer is the leading cause of cancer death worldwide with an estimation of 1.59 million deaths per year corresponding to 19.4% of all cancer associated deaths [1]. Most lung cancers do not exhibit specific symptoms and are often detected at an advanced stage of the disease. In smokers, the latent period of lung cancer is estimated as at least 20 years [2]. Only 15.6% of lung cancers are diagnosed at an early localized stage I or II [3], and 65.9% fall within stages III and IV [4]. Patients with a diagnosis of stage I lung cancer have a survival rate of 71%.

Low-dose spiral computed tomography (LDCT) scan is currently being used to screen a high risk population of heavy smokers [5]. The results of LDCT screenings appear to be more relevant when restrictive criteria, mainly involving smoking habit and age, for high-risk population are applied; consequently, part of the population is excluded from current CT screening programs. Furthermore, the high rate of false positives leads to multiple follow-up examinations and often to unnecessary surgery [6] while repeated CT scans cause excessive irradiation, involve high costs and are therefore not ideal for preventive screening in general population [7]. Nevertheless, LDCT screening remains the cost-effective approach for high-risk population [8]. There is a need to complement LDCT with the method which can be applied to extended low-risk population. Non-invasive biomarker tests for detection of lung cancer may become such an alternative and its results may be used to recommend further LDCT diagnostics. The combination of these two approaches may result in more accurate cancer detection.

Blood cancer biomarkers have been reported based on gene expression [9], genetics and epigenetics of circulating free DNA [10], miRNAs [11], proteins [12], and auto-antibodies [13]. Autoimmune antibodies are particularly promising, as altered proteins produced in cancer cells generate tumor-specific antigens that elicit a host immune response. Significant body of evidence exists for the presence of circulating antibodies to autologous tumor-associated antigens (TAAs) in blood serum samples from patients with different cancers, including lung cancer [14–20].

Several TAAs have been identified and used as serum markers for the early diagnosis of lung cancer. Many TAAs, such as P53, HER2, CEA, CAGE, Annexin 1, SOX2, or MUC1 are involved in essential cellular functions, including DNA replication, transcription regulation, mRNA splicing and translation [14,21–24]. However, none of these biomarker performances reaches sufficient sensitivity and specificity for application as screening markers for lung cancer detection.

Immunogenic potential of the tumor suppressor BRCA1-associated RING domain 1 (BARD1) has been shown in a screen for antigens protecting against experimentally induced cancer in mice [25]. BARD1 is a major binding protein of the breast cancer predisposition gene product BRCA1 [26,27]. Bound to BRCA1, BARD1 is an essential component of BRCA1’s tumor suppressor activity due to the E3 ubiquitin ligase activity of the BRCA1-BARD1 heterodimer [28]. Independently of BRCA1, BARD1 is an inducer of apoptosis by binding to and stabilizing p53 [29–31]. In recent years, truncated and deletion-bearing BARD1 isoforms, generated through alternative splicing of the BARD1 gene, have been discovered in various cancers and their expression correlated with disease progression and poor prognosis [32–38]. These isoforms, abundantly expressed in tumors and cancer cell lines, lack the BRCA1-interacting N-terminal RING domain and do not retain tumor suppressor functions but have oncogenic potential [32,39,40]. The over-expression of BARD1 isoforms was strongly correlated with tumor progression, specifically in non-small-cell lung cancer (NSCLC) [37]. The different exon combinations of isoforms suggest that they acquire a different tertiary structure. It is therefore conceivable that BARD1 isoforms present TAAs and consistent with the previously reported immunogenicity of BARD1 [25]. As BARD1 isoforms are tell tales of tumor progression and TAAs, we thought to develop a blood test for the early detection and diagnosis of lung cancer based on capturing autoimmune antibodies against BARD1 antigens.

## Materials and methods

### Serum samples

Blood serum samples were collected in four different centers: University Hospitals of Geneva (Switzerland), University of Molise (Italy), Medical University of Vienna (Austria), and National Koranyi Institute of Pulmology, Budapest (Hungary). Informed consent for the scientific use of biological material was obtained from all patients and healthy blood donors in accordance with the requirements of the local ethics committees of the involved institutions. Sera from 93 consecutive chemotherapy-naive patients with non-small cell lung cancer (NSCLC) and 94 healthy controls were included in the modeling. Information on gender, age and diagnosis of lung cancer type and stage is provided (Table 1).

**Table 1:** Clinical characteristics of lung cancer and control study populations.

Samples	Characteristics	Lung Cancer	Control
**Total**		93	94
**Gender**	Females	36	41
	Males	57	34
	Unkown	0	19
**Age**	Range	28–86	19–85
	Median	65	54
**Histology**	NSCLC nonspecified	28	
	Adenocarcinoma	42	
	Large cells	1	
	Squamous cell carcinoma	22	
**Stage**	IA	0	
	IB	2	
	IIA	3	
	IIB	4	
	IIIA	22	
	IIIB	16	
	IV	39	
	Unknown	7	

Additional sera from patients with other cancers than lung cancer (Table 2) comprised 81 serum samples from patients with breast cancer, ovarian cancer, and neuroblastoma.

**Table 2:** Clinical characteristics of breast cancer, neuroblastoma and ovarian cancer study populations.

Samples	Characteristics	Breast Cancer	Neuroblastoma	Ovarian Cancer
**Total**		12	20	14
**Gender**	Females	12		14
	Males	0		0
	Unkown	0	20	0
**Age**	Range			38–82
	Median			57

### Antigen selection

**Fig 1:**
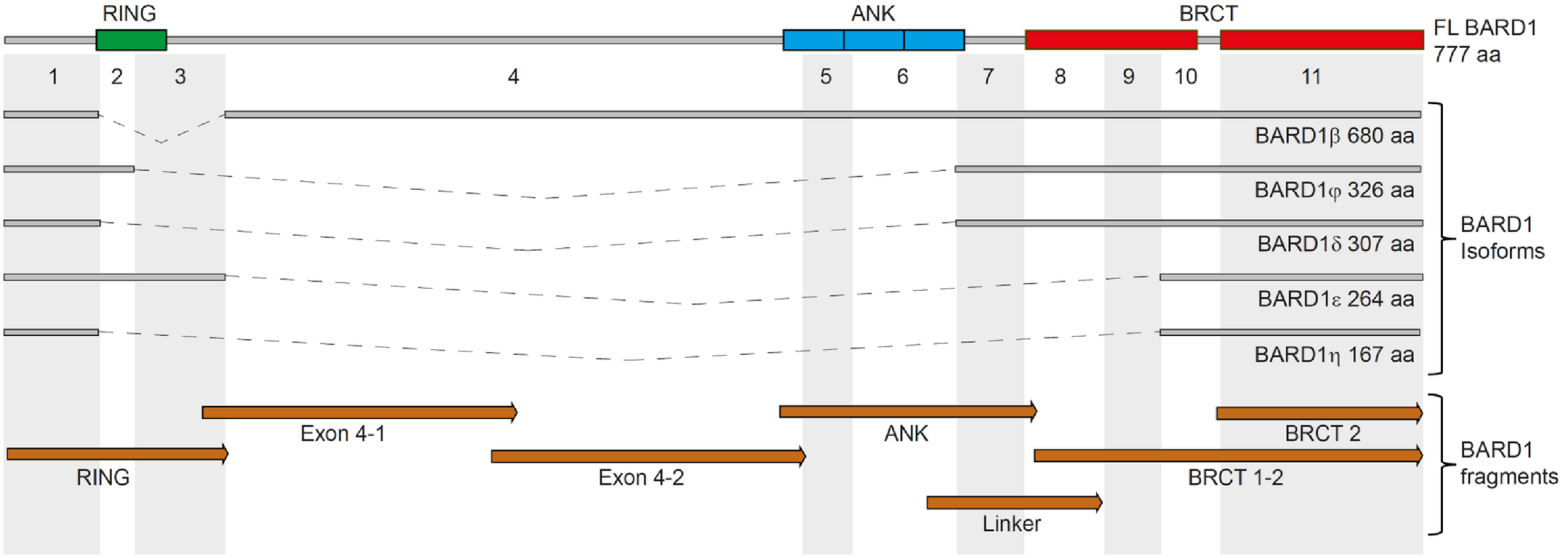
BARD1 protein structure and epitopes. The top line shows FL BARD1 exon structure is shown with protein motives RING, Ankyrin (ANK) repeats, and BRCT domains indicated. Grey lines underneath show BARD1 isoforms with dotted lines representing the respective missing exons. Brown bars on the bottom represent protein fragments used for ELISA experiments.

We designed a library of 33 peptides of 10 to 20 amino acids in length chosen from predicted antigenic sites of the protein sequences of FL BARD1 or BARD1 isoforms (Fig 1). GeneScript OptimumAntigen antigen design tool was used to predict immunogenic epitopes in the sequences of BARD1 (Uniprot accession Q99728), BARD1β (Gene Bank accession NP_001269472), BARD1δ (Uniprot accession F6MDI1), BARD1ϕ (Uniprot accession F6MDI0), BARD1ε (Uniprot accession F6MDI2) and BARD1η (Uniprot accession F6MDI3). The epitopes were chosen to represent each BARD1 exon or cover the exon junctions specific for BARD1 isoforms listed above (S1 Fig, S1 Table). Peptides were synthesized at 95% purity (Genscript). A small number of peptides showed only insignificant antibody binding activity in tests with known BARD1 antibodies and was excluded for further tests.

We also generated seven histidine-tagged fusion proteins representing sub-fragments of BARD1 of 129 to 246 amino acids length and covering the entire BARD1 protein sequence (S1 Fig, S1 Table). These BARD1 protein fragments were produced in E. Coli BL21* and purified using Ni-NTA agarose (QIAGEN) under denaturing conditions.

### ELISA assays

For ELISA assays peptides and fragments were custom spotted onto wells of 96-well plates by the plate manufacturer MSD (Meso Scale Discovery, Rockville, MD) at approximately 0.05μg per spot. Antigens were incubated with serum samples from cancer patients and healthy controls and MSD electrochemiluminescence assays were performed according to the manufacturer’s specifications. Plates were incubated with PBS-5% Blocker A solution for 1 h at room temperature. Blocking solution was discarded and 25 μl of serum samples diluted at 1:200 in PBS-1% Blocker A were added and incubated for 2h at RT. After washing with PBS-0.05%Tween, 25 μl of anti-human SULFO-TAG detection antibody (# R32AJ-5, Meso Scale Discovery) diluted 1.2 μg/ml in PBS 1% Blocker A was added and incubated for 1h at RT. After three PBS-0.05% Tween washes, 150 μl 2xMSD Read Buffer T was added to the wells and plate reading was immediately performed on the Meso Scale Discovery Sector Imager 2400. To minimize experimental variability, cancer and control samples were distributed at equal proportion on each plate. Each plate contained wells probed with known anti-BARD1 antibodies against epitopes that were tested previously in various studies [37–39,41]. Only sera that were assayed with each of the 40 antigens tested were included in the statistical analysis and in Tables 1 and 2. The mean values for each sample measurements were used for the modelling.

### Statistical analysis

To achieve efficient discrimination between cancer and control patients, we performed logistic regression analysis using the Lasso (least absolute shrinkage and selection operator) method [42–44]. All computations and analyses were performed using the R software. The glmnet package [42] was used for fitting lasso logistic regression, and the ROCR [45] and OptimalCutpoints [46] packages were used for generating and analyzing ROC curves.

## Results

### Detection of serum autoantibodies against BARD1 antigens in lung cancer patients

Multiple BARD1 isoforms are overexpressed in various cancer, with a particular combination of isoforms in lung cancer, while the expression of full length (FL) BARD1 is absent or reduced [35,37]. As isoforms of BARD1 have internal deletions, they are likely to fold differently than FL BARD1 and present tumor antigens. To generate tools for autoantibody capturing, we synthesized peptides that represent the predicted most immunogenic epitopes from the protein sequence encoded by the 11 exons of FL BARD1, as well peptides covering exon junctions unique for isoforms (Fig 1). We also generated seven recombinant BARD1 protein fragments, which together represent the entire FL BARD1 protein (Fig 1).

**Fig 2:**
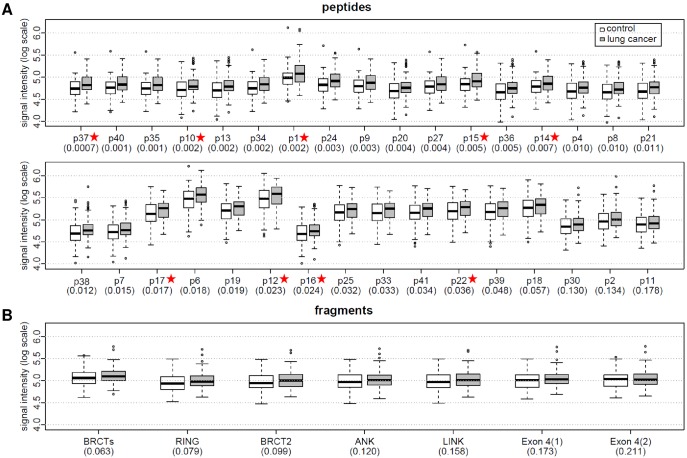
Comparison of ELISA signal intensities of controls and lung cancer patients using peptides and protein fragments for capturing anti-BARD1 autoimmune antibodies. (A) Log transformed signal intensities for peptides are presented in increasing order of their p-values obtained applying the Wilcoxon’s rank-sum test from left to right. For 15 peptides the resulting p-values were lower than 0.01, and for 29 peptides lower than 0.05. The signal intensities tend to be significantly higher for lung cancer samples than for controls. Ten peptides scored highest in 27 peptides model and common with 18 peptides model are marked with red stars. (B) Log transformed signal intensities of fragments are presented aligned in increasing order of their p-values from left to right.

To determine the presence of anti-BARD1 antibodies in cancer patients, we tested sera from patients with lung cancers and controls (Table 1) with 33 peptides and seven BARD1 fragments in ELISA assays. The analysis of the readout data showed in average higher signals for serum samples from cancer patients than from controls, but high variability of antigen signals for both the cancer patient and the control serum samples (Fig 2). A one-sided Wilcoxon’s rank-sum test was performed for each antigen to determine if the signal differences between cancer and control samples were statistically significant. For the majority of antigens (29 out of 40), the resulting p-values were lower than 0.05, and for 15 peptides lower than 0.01. Thus our data provided statistical evidence that the majority of the antigens generated higher values in the cancer samples than in controls.

### Discrimination between lung cancer and healthy controls

**Fig 3:**
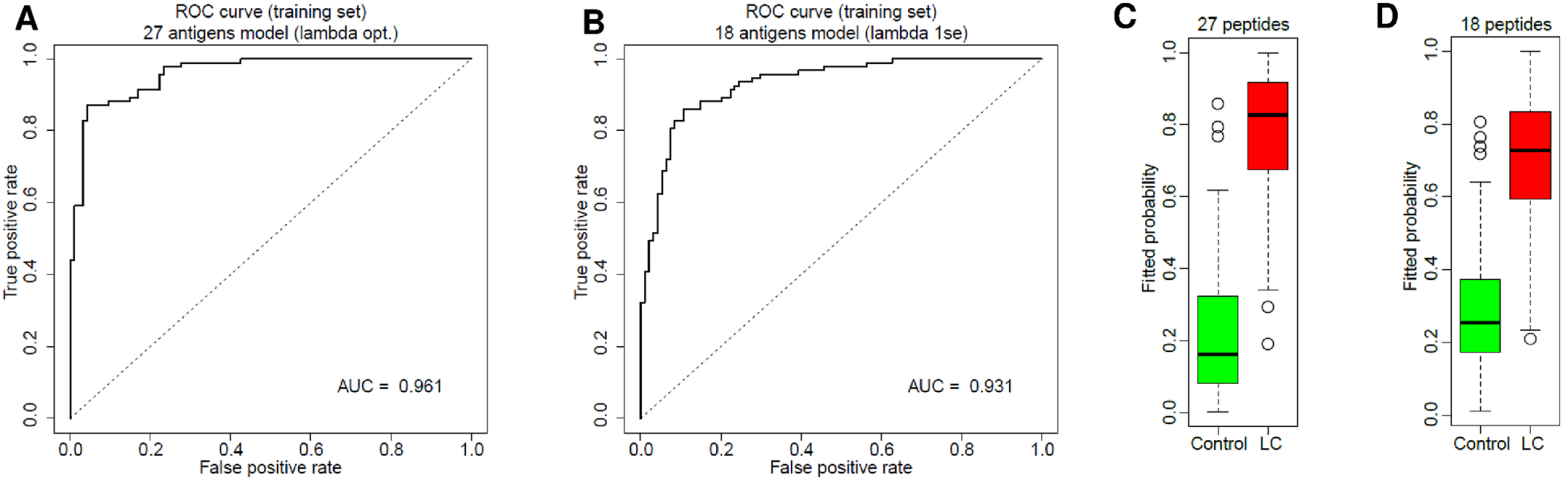
Fitted lasso logistic regression for distinction between lung cancer and controls. (A) ROC curve and AUC value for prediction on the whole sample (= training set) is shown for the optimal model based on 27-peptides (λ_min_), AUC = 0.961. (B) ROC curve of the second-best 18-peptide model is shown (λ_1se_), AUC = 0.931. (C-D) Fitted scores (estimation of the probability that the patient has lung cancer) are shown for 27-peptide model (C) with 18-peptide model (D).

Although the signal intensities were significantly higher for patients with lung cancer than for control cases, no single individual antigen signal alone was sufficient for discriminating lung cancer from controls with high sensitivity and specificity, due to the high variability. Therefore we built prediction models for discriminating lung cancer from controls based on their autoantibody profiles applying the Lasso method [42]. The best model obtained consisted of 27 antigens (S2 Table) (predictors) which yielded a Receiver Operating Characteristic (ROC) curve with the Area Under the Curve (AUC) = 0.96 (Fig 3A). To determine the impact of the number of predictors, we defined a model with 18 peptides (S2 Table), which also results in a high AUC = 0.93 (Fig 3B). These results show that both models discriminate efficiently lung cancer samples from healthy controls and that increasing the number of predictors does not considerably improve the AUC (Fig 3C and 3D). The high AUC values obtained with our models present evidence for a sufficient capacity of the BARD1 autoimmune antibody-based test for lung cancer detection with high specificity and sensitivity.

To further develop clinically applicable lung cancer diagnostic test, it is important to reduce the number of predictors to be used. We have selected ten peptides: p37, p13, p10, p17, p12, p14, p15, p16, p22 and p1 (Fig 2A). These antigens were scored highest in the 27 peptides model and in common with 18 peptides model. The selected predictors will be evaluated using independent patients cohorts.

**Fig 4:**
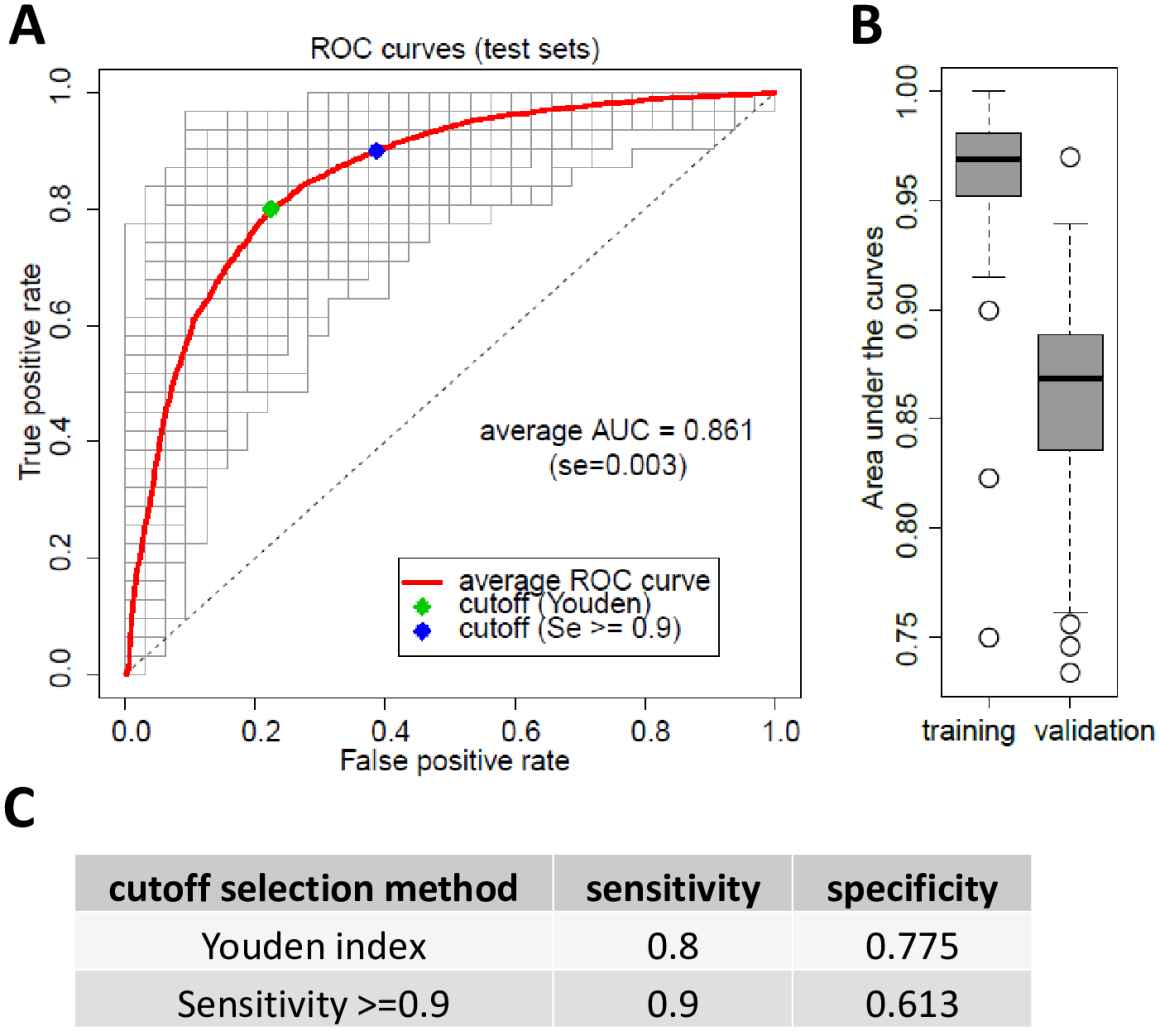
Validation of BARD1 lung cancer test by random repeated splitting of samples in training and validation sets. (A) All samples were 200 times randomly split in training sets and validation sets and modeling was performed on training sets and applied on validation sets. The distribution of the respective AUCs obtained for 200 training sets and validation sets is presented. (B) The 200 ROC curves for prediction on the validation sets are shown, always consisting of 31 lung cancer and 32 control patients. The average ROC curve is plotted in red. The average AUC for these validation sets was 0.861 (se = 0.003). The points on the average ROC curve correspond to the optimal cutoff obtained by the Youden’s method and the one obtained with a sensitivity higher than 0.9. (C) The sensitivity and specificity are provided for both methods of optimal cutoff selection.

In order to validate the above described models, we split the samples in test and validation sets in order to validate the discriminative capacity of our modeling approach for independent samples. To evaluate the classification ability and the variability of our model we repeatedly and randomly split the samples into “training” sets comprising about 66% of the samples (62 lung cancer and 62 control samples) and “validation” sets comprising 34% of the samples (31 lung cancer and 32 controls). We created 200 pairs of randomly split training/validation sets. The 200 training sets were used to build 200 different discriminative models, which were then applied on the 200 corresponding validation sets. This procedure yielded average AUC on training sets of 0.96 (SE = 0.002), similar to results obtained with the whole sample set. The average ROC curve for the validation sets was AUC = 0.86 (Standard Error (SE) = 0.003) (Fig 4A and 4B).

To define how these models would apply as a lung cancer test, we determined a cutoff in order to optimize the ratio between specificity and sensitivity. To do this, we applied two methods using the average ROC curve of the validation sets in Fig 4B. The first one was to maximize the vertical distance between the ROC curve and the diagonal curve (Youden index), which provides an optimal specificity/sensitivity ratio (Fig 4C). This approach resulted in a sensitivity of 0.80 and specificity of 0.78. The second method was to choose a cutoff at maximal specificity for minimal sensitivity chosen at 0.9, which provided a specificity of 0.61 (Fig 4C). Detecting combined stages 1–3 and late stage 4 lung cancers

We addressed the question of whether a Lasso logistic model yields different sensitivities depending on the stage of lung cancer. Our sample collection comprised 47 samples from lung cancer patients with stages IB, IIA, IIB, IIIA, and IIIB, 39 at stage IV, and 7 of unknown disease status (Table 1). Due to the small sample size, it was not possible to make sub-groups of samples from stage I or II lung cancer individually, but we grouped the samples in early (I-II) or limited (III) disease and late (IV) or metastatic stage.

**Fig 5:**
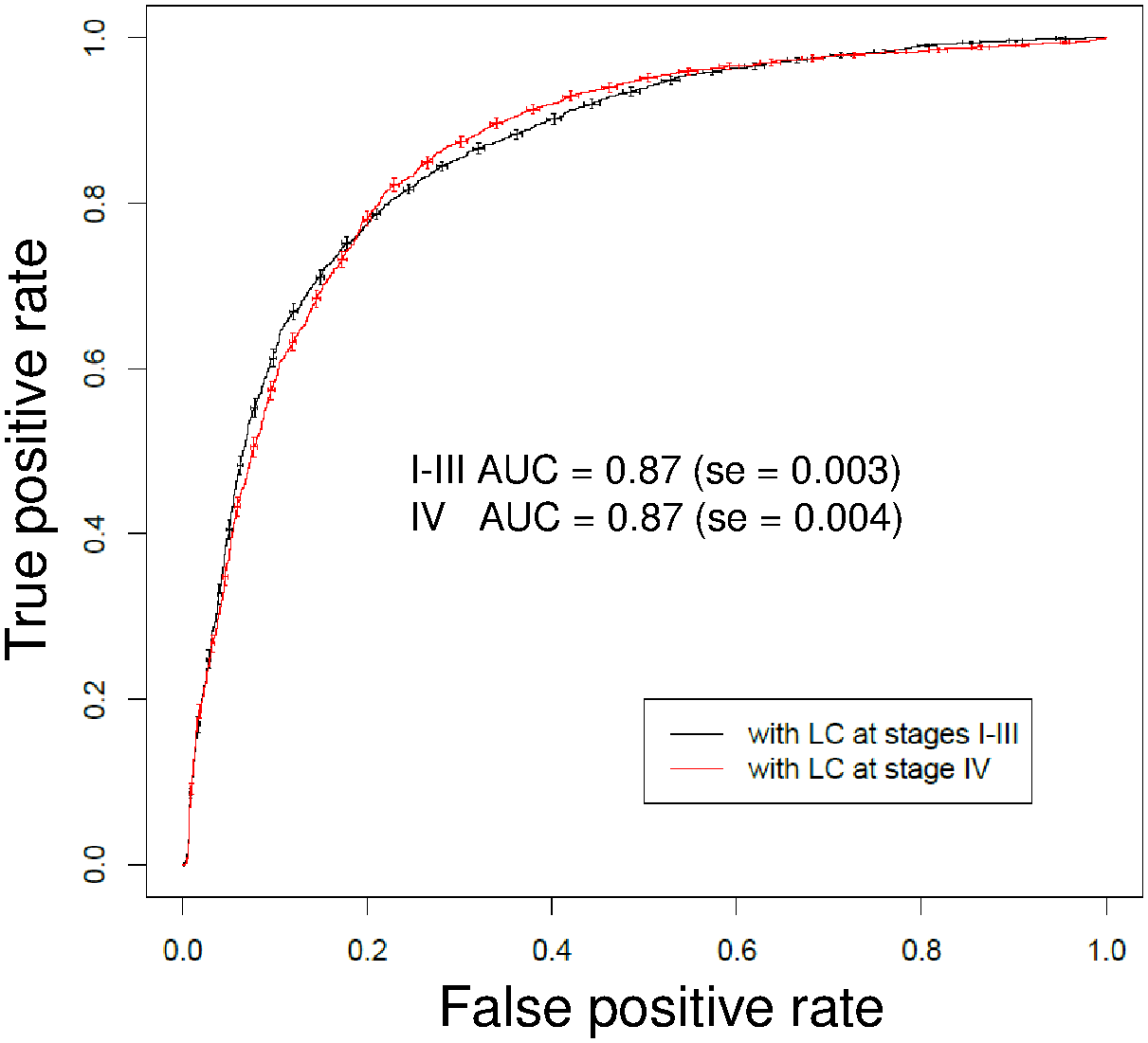
Comparison of ROC curves for in early or limited disease and late lung cancer. During each of the 200 repetitions of random sub-sampling validation (Fig 4), the lung cancer patients of the training set were divided into two groups according to stage of the disease I-III and IV and 200 ROC curves were computed for each group separately. The average ROC curves are shown and respective AUCs. The error bars indicate ± 1 standard error of the mean.

We performed 200 repetitions of random sub-sampling in training and validation sets as shown in Fig 4. The results are presented as two different average ROC curves. These average ROC curves had an AUC = 0.87 for stages I-III and AUC = 0.87 for stage IV (Fig 5). These nearly identical curves indicate that the probability for the detection of early or limited disease stage lung cancer is as high as for advanced stage cancer with the BARD1 autoimmune antibody-based test.

### The BARD1 lung cancer test is specific for lung cancer

The BRCA1 and BARD1 genes and proteins play crucial role in the development of various cancers other than lung cancer. Furthermore, BARD1 isoforms have been described in particular in breast and ovarian cancers, as well as neuroblastoma, although with a cancer-type specific pattern of expression [32,33,47,48].

**Fig 6:**
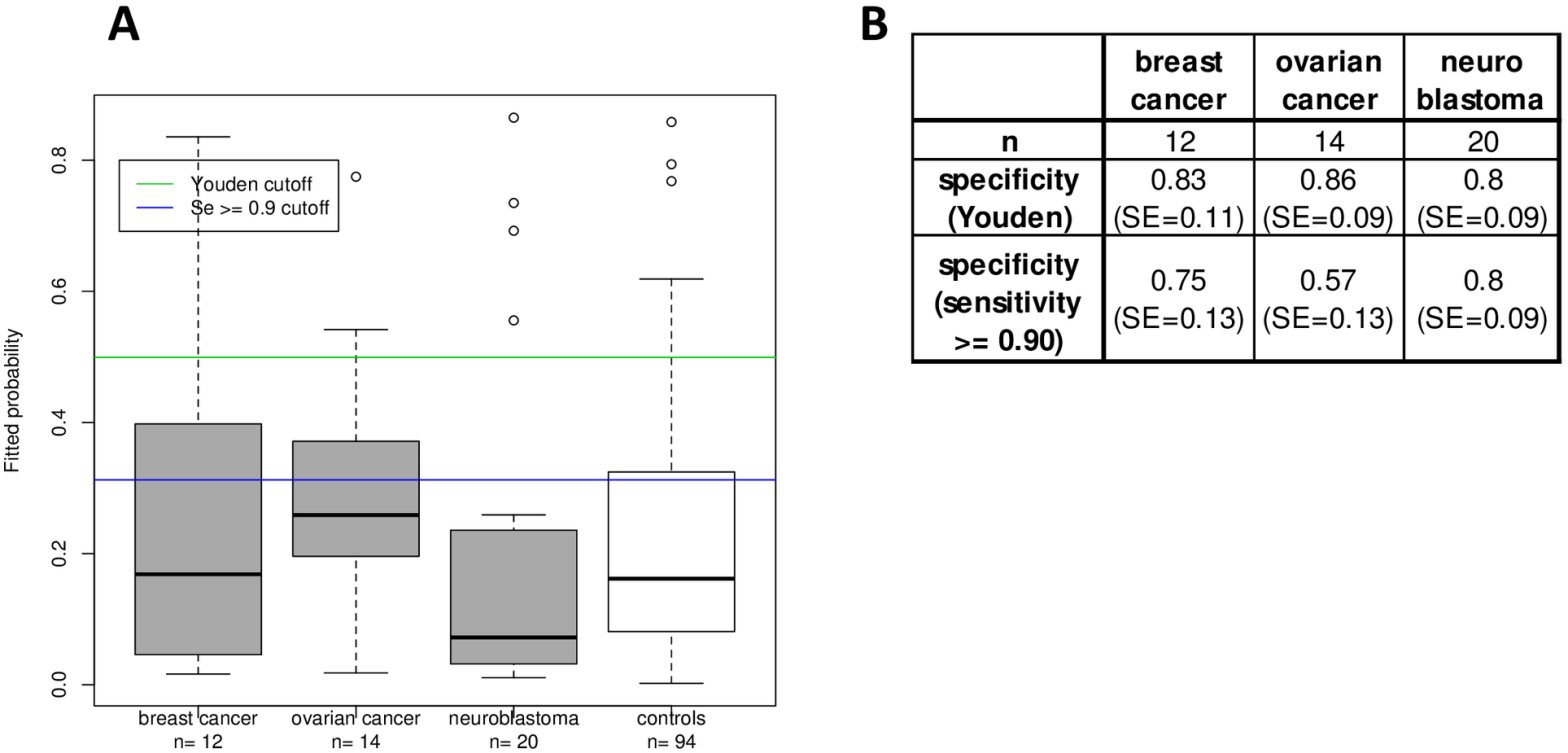
Specificity of the 27-antigens model in regard of detection of other types of cancers. (A) The final 27-antigens model was applied for prediction on patients with breast or ovarian cancer, neuroblastoma or controls. The estimated probability that these patients are identified as positive with the lung cancer model are plotted. The two horizontal lines indicate the optimal cutoffs obtained by applying the Youden index and the method of Sensitivity at least 0.9 (SE>0.9) on the average ROC curves for the validation sets presented in Fig 4B. At these cutoffs, only few of the breast and ovarian cancer cases are falsely detected as lung cancer, but none of neuroblastoma cases. These results demonstrate that a panel of BARD1 antigens can be used in a blood test for the specific detection of lung cancer with high sensitivity and specificity.

We applied the Lasso fitted 27-antigen model on test results for sera from breast and ovarian cancer and neuroblastoma patients (Table 2) to determine the BARD1 lung cancer test specificity in respect of possible cross-reactivity with other cancers. Applying the model, the results showed little cross-reactivity of the BARD1 lung cancer test with the three types of cancer (Fig 6A). To predict positivity, we determined cutoffs applying the Youden’s method and the optimal cutoff with a sensitivity of at least 0.9 (Fig 6A). For both cutoffs only few breast and ovarian cancer and neuroblastoma samples were falsely detected with the lung cancer model. However, the values for specificity are comparable to values obtained for testing lung cancer versus control samples, namely between 0.80 and 0.86 (Fig 6B).

## Discussion

Worldwide, 2 million people are diagnosed with lung cancer every year, and 1.6 million die from lung cancer; this makes lung cancer the most deadly cancer. Many of these deaths could be avoided if lung cancer would be diagnosed at an earlier stage when available treatments are still effective. To be most efficient, such a test should be highly sensitive, reliable, easy to perform, affordable, and should be repeated in yearly intervals to monitor the at-risk populations. Previous publications have shown that autoantibodies to tumor-associated antigens are useful biomarkers for the detection of early-stage lung cancers [49].

### BARD1 not just a lung cancer biomarker, but driver of tumorigenesis

In recent years the number of potential biomarkers for the early detection of lung cancer has increased. High throughput technological advances such as mutation analysis, deep sequencing, and genome wide association studies, as well as proteomic analysis have identified a large number of possible markers for lung cancer. Novel predictive and prognostic molecular markers in non small- cell lung cancer (NSCLC) include DNA damage repair genes, such as ERCC1, RRM1 and BRCA1 [50]. Upregulated expression of BRCA1 mRNA was correlated with reduced survival of NSCLC patients and presented as predictive biomarker for response to treatment [51,52]. TAA antigens present a very promising approach towards a test for early detection of lung cancer [49]. Unfortunately, none of the published biomarker tests showed very high sensitivity for lung cancer detection.

We report here that autoantibodies against the tumor suppressor BARD1 are useful biomarkers for the detection of lung cancer resulting in a lung cancer test with high sensitivity and specificity.

Antibodies against cancer-associated isoforms of BARD1 can be detected in the serum of lung cancer patients. BARD1 isoforms have been associated with lung cancer progression [35,37]. The expression level of BARD1 isoforms correlated with decreased disease free survival and overall survival of NSCLC patients and with tumor progression in an animal model of lung cancer. These results demonstrated that BARD1 isoform expression reflects tumor progression, consistent with the confirmed role of some BARD1 isoforms as drivers of tumorigenesis [32,39]. Therefore autoimmune antibodies against BARD1 in lung cancer patients are reflect the existence of tumorigenic BARd1 isoforms and are tell tales of cancer.

### Lung cancer-specific autoimmune BARD1 antibodies signature

BARD1 epitopes were first described as tumor antigens in a screen for tumor antigens in a murine mouse model [25].

Tumors express BARD1 isoforms while the expression of FL BARD1 is down-regulated. The cancer specific BARD1 isoforms with diverse tertiary structures might present immunogenic BARD1 epitopes. However, the isoform pattern and hence the generation of reactive antibodies might be variable from one patient to another. Therefore we aimed to define an autoantibody signature able to discriminate cancer patients from healthy controls.

There are a number of different classification algorithms available, each with their own strengths and limitations. When analyzing a small dataset stepwise covariate modelling procedure (SCM) may produce a covariate model that suffers from selection bias and poor predictive performance. Therefore we performed Lasso logistic regression for efficient discrimination between cancer and control patients along with the selection of a limited number of predictors.

This model applied to the whole data set predicts that our test could distinguish lung cancer from healthy controls and from other cancers with high sensitivity and specificity and an AUC = 0.96. We validated the model by splitting the samples randomly and repeatedly 2 to 1 in training sets and validation sets, which resulted in average ROC curve for validation sets with an AUC = 0.86. Applying the model on in early (I-II) or limited (III) disease and late (IV) stage cancer resulted in identical ROC curves with an AUC = 0.87. These data suggest that the BARD1 autoimmune antibody test could be a potent early detection test for lung cancer. To validate further the ability of our test system to discriminate early stage (I-II) lung cancers from controls we plan to increase early stage cohort size to obtain statistically significant evaluation. From our results we also conclude that increasing the sample size will lead to higher AUCs even for the validation sets.

We demonstrate that the autoantibody signature is specific for lung cancer, but specific optimized signatures could potentially be developed for subgroups of patients (e.g. females and males, NSCLC, SCLC) and might result in even better sensitivity and specificity.

Autoantibodies are potentially well suited as cancer biomarkers, because only a minimal-invasive intervention is needed for their extraction, they can be easily measured, they are stable in blood, and they have a long half-life. The drawback of single autoantibodies is their low diagnostic sensitivity. Here, we show that a panel of BARD1 peptides for the creation of complex autoantibody profiles allows the differentiation of lung cancer patients from healthy blood donors with high accuracy.

## Supporting information

S1 Fig BARD1 peptides antigens. (PDF)

S1 Table BARD1 antigens amino acid sequences. (XLSX)

S2 Table 18 antigens and 27 antigens models coefficients. (PDF)
