# Supplementary material for: BARD1 serum autoantibodies for the detection of lung cancer
Source: PLoS One. 2017 Aug 7;12(8):e0182356. doi: 10.1371/journal.pone.0182356 (PMC5546601; doi:10.1371/journal.pone.0182356)
Supplement: S1 Fig — (PDF) [file pone.0182356.s001.pdf]

S1 Figure. BARD1 peptides antigens

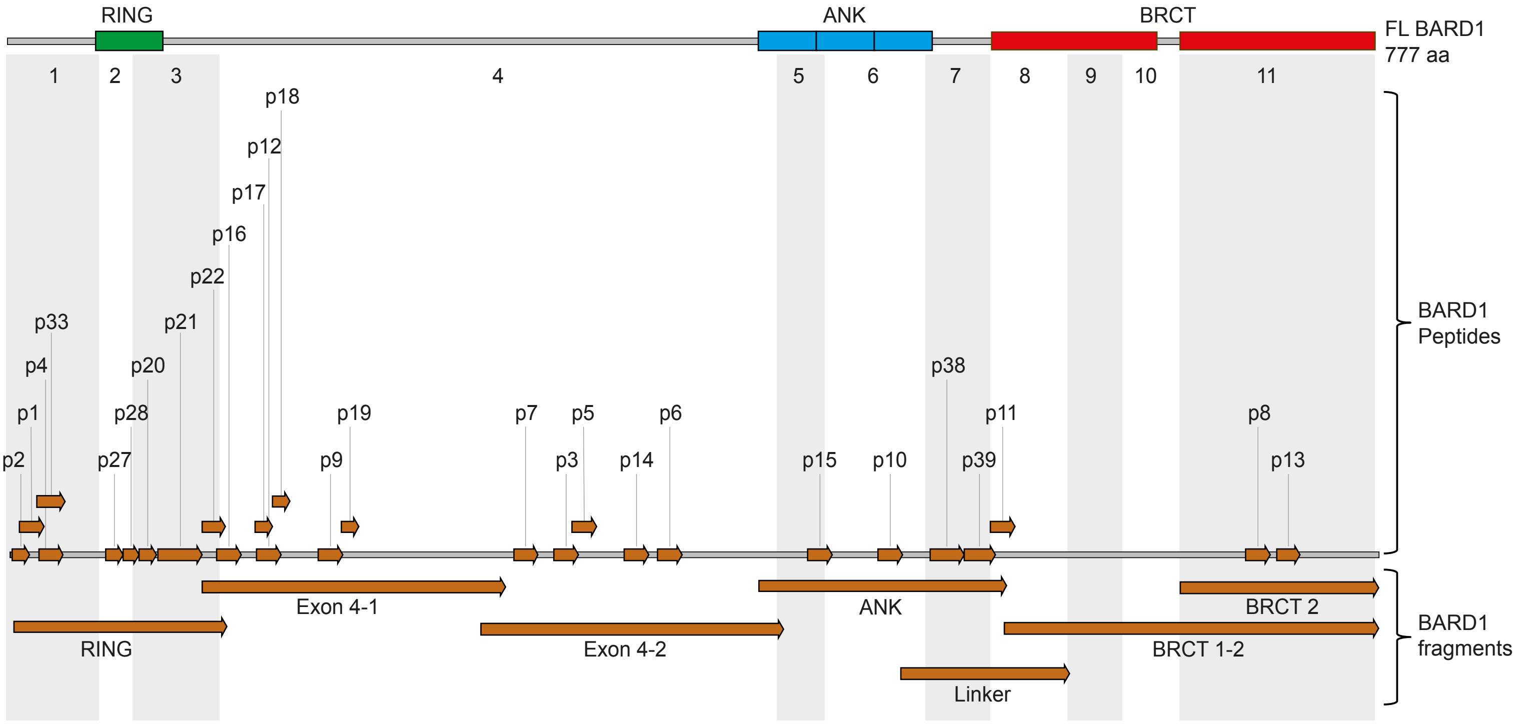

p24 p25 p26 BARD1β alternative ORF
p30 BARD1β exons 1/4 junction
p37 BARD1φ exons 2/7 junction
p34 BARD1δ exons 1/7 junction
p36 BARD1ε exons 3/10 junction
p35 BARD1η exons 1/10 junction

} Isoform-specific peptides

The positions of peptides antigens relative to BARD1 protein sequence are shown on scheme for the peptides mapped to full-length BARD1 sequence. The peptides specific to the BARD1 isoforms are described at the bottom.
