# Supplementary material for: BARD1 serum autoantibodies for the detection of lung cancer
Source: PLoS One. 2017 Aug 7;12(8):e0182356. doi: 10.1371/journal.pone.0182356 (PMC5546601; doi:10.1371/journal.pone.0182356)
Supplement: S2 Table — (PDF) [file pone.0182356.s003.pdf]

S2 Table. 18 antigens and 27 antigens models coefficients

18 antigens model	
**Peptide**	**Coefficient**
(Intercept)	-17.7
p37	20.7
p13	20.2
p10	16.1
p12	2.7
BRCT 2	1.9
p2	1.6
BRCT 1-2	1.3
p4	1.2
p17	0.8
p36	0.2
p33	0.0
Exon 4-1	-0.5
p22	-1.8
p1	-2.7
Exon 4-2	-3.7
p14	-7.8
p15	-8.7
p16	-38.1

27 antigens model	
**Peptide**	**Coefficient**
(Intercept)	-17.4
p37	33.1
p13	28.1
p10	24.8
p17	10.9
p12	9.4
p35	7.6
p2	4.0
p36	3.9
p4	2.9
BRCT 1-2	2.5
BRCT 2	2.2
p19	1.4
RING	1.0
ANK	0.7
LINK	-0.1
p11	-0.5
p1	-2.2
Exon 4-1	-2.6
p38	-2.9
p22	-4.9
p6	-6.0
p7	-6.2
Exon 4-2	-6.4
p33	-9.5
p14	-16.1
p15	-20.2
p16	-51.1
